# *Cynanchum atratum* Alleviates Non-Alcoholic Fatty Liver by Balancing Lipogenesis and Fatty Acid Oxidation in a High-Fat, High-Fructose Diet Mice Model

**DOI:** 10.3390/cells11010023

**Published:** 2021-12-22

**Authors:** Jing-Hua Wang, Seung-Ju Hwang, Dong-Woo Lim, Chang-Gue Son

**Affiliations:** 1 Institute of Bioscience & Integrative Medicine, Daejeon University, 75, Daedeok-daero 176, Seo-gu, Daejeon 35235, Korea; bluesea9292@naver.com; 2 Liver and Immunology Research Center, Daejeon Korean Medicine Hospital, 75, Daedeok-daero 176, Seo-gu, Daejeon 35235, Korea; 3 Department of Diagnostics, College of Korean Medicine, Dongguk University, Dongguk-Ro 32, Goyang 10326, Korea; greatwoodong@dongguk.edu; 4 Institute of Korean Medicine, Dongguk University, Dongguk-Ro 32, Goyang 10326, Korea

**Keywords:** herbal medicine, NAFLD, high-fat diet, high-fructose diet, AMPK, free fatty acid

## Abstract

*Cynanchum atratum*, a medicinal herb, is traditionally used as an antidote, diuretic, and antipyretic in eastern Asia. The current study aimed to investigate the anti-fatty liver capacity of the ethanol extract of *Cynanchum atratum* (CAE) using a 10-week high-fat, high-fructose diet mouse model. A six-week treatment of CAE (from the fifth week) significantly attenuated the weights of the body, liver, and mesenteric fat without a change in diet intake. CAE also considerably restored the alterations of serum aminotransferases and free fatty acid, fasting blood glucose, serum and hepatic triglyceride, and total cholesterol, as well as platelet and leukocyte counts. Meanwhile, CAE ameliorated hepatic injury and lipid accumulation, as evidenced by histopathological and immunofluorescence observations. Additionally, CAE significantly lowered the elevation of hepatic TNF-α, the TNF-α/IL-10 ratio, fecal endotoxins, and the abundance of Gram-negative bacteria. Hepatic lipogenesis and β-oxidation-related proteins and gene expression, including PPAR-α, SREBP-1, SIRT1, FAS, CTP1, etc., were normalized markedly by CAE. In particular, the AMPK, a central regulator of energy metabolism, was phosphorylated by CAE at an even higher rate than metformin. Overall, CAE exerts anti-hepatic steatosis effects by reducing lipogenesis and enhancing fatty acid oxidation. Consequently, *Cynanchum atratum* is expected to be a promising candidate for treating chronic metabolic diseases.

## 1. Introduction

The metabolism is a fundamental basis of life in all living organisms [1]. In the past, genetic defects were the primary cause of metabolic disorders [2]. However, in recent decades, the prevalence of chronic metabolic diseases, including non-alcoholic fatty liver disease (NAFLD), has increased rapidly due to extensive changes in the modern lifestyle and external environment [3]. It is worth noting that, at present, approximately a quarter of the worldwide population has NAFLD due to the pandemic spread of chronic metabolic dysfunctions, including obesity and hyperlipidemia [4]. Asia has presented a higher prevalence than Western countries in recent years, which has resulted in a heavy economic burden and a mortality increase in Asia [5]. However, the heterogeneous pathogenesis of NAFLD is multifaceted, and is characterized as “multiple hits to multiple organs”, which, up to now, is still hard to describe [6]. Effective drugs or therapies for NAFLD have not been officially approved in Western medicine yet [7]. Therefore, studies are emerging that explore viable strategies to alleviate NAFLD from various perspectives. 

Recently, a great deal of preclinical evidence revealed that natural herbs exert an anti-hepatosteatosis effect through a variety of approaches and pathways [8,9], such as lipogenesis inhibition [10], the augmentation of fatty acid oxidation [11], and the regulation of gut microbiota [12], etc. In addition, according to the data from ClinicalTrails.gov, an increasing number of clinical trials related to NAFLD/NASH using herbs or their active compounds are being registered [13]. However, most of the herbs and active compounds studied are used as dietary supplements rather than drugs [13]. Thus, the discovery of potent herbal medicine candidates and the clarification of the corresponding molecular mechanisms are urgently required to advance the fight against NAFLD.

In eastern Asia, *Cynanchum atratum* is traditionally used as an antifebrile, diuretic, antidote, and antiphlogistic to improve various ailments, e.g., asthenic fever, rheumatologic diseases, urinary infections, and scrofula, etc. Many in vivo and in vitro studies have also indicated that *Cynanchum atratum* and its derived compounds ameliorate respiratory inflammation [14], atopic dermatitis [15], mastitis [16], melanogenesis [17], parasitosis [18], tuberculosis [19], acute liver injury [20], and leukemia [21]. However, whether *Cynanchum atratum* can improve diet-induced metabolic dysfunctions, including non-alcoholic fatty liver disease, remains unclear.

On account of the long-term clinical application and diverse pharmacological activities of *Cynanchum atratum*, the anti-fatty liver efficacy of *Cynanchum atratum* was investigated herein using a high-fat and high-fructose diet (HFHFD) mouse model. We also attempted to examine the potential molecular mechanisms as a basis for the next step of drug development.

## 2. Materials and Methods

### 2.1. Preparation of Cynanchum Atratum Extraction

**Figure 1:**
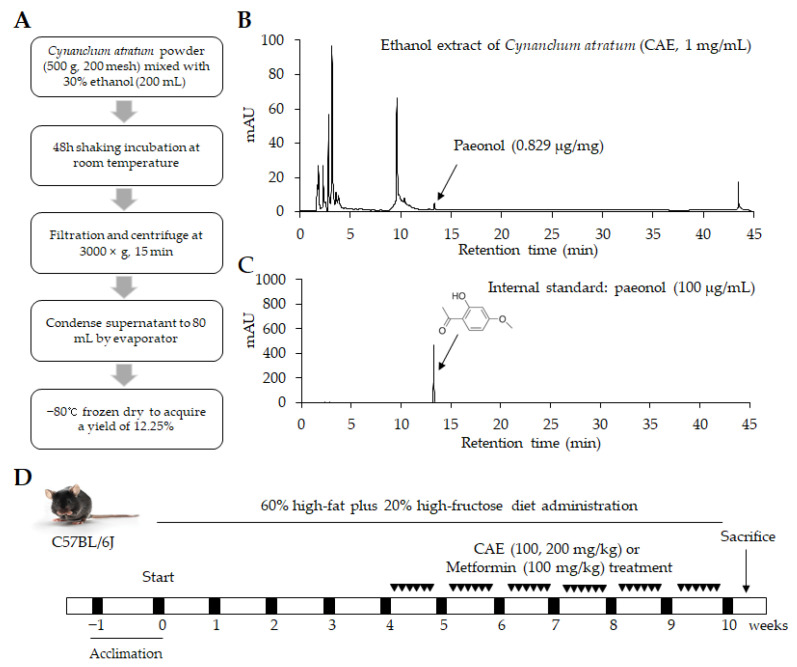
Herbal extract and design of the animal study. (**A**) The brief procedure of *Cynanchum atratum* extraction (CAE). HPLC-based fingerprinting of (**B**) 30% ethanol extract of *Cynanchum atratum* (CAE) and (**C**) paeonol (internal standard) were determined at a wavelength of 300 nm. A synthetic scheme presents the information regarding experimental design (**D**).

*Cynanchum atratum* was purchased from the Jeong-Seong Pharmaceutical Company (Daejeon, Korea). The ethanol extract of *Cynanchum atratum* (CAE) was produced as follows: 500 g of dried *Cynanchum atratum* was ground into a powder and then mixed with 200 mL of 30% ethanol at room temperature (RT) and underwent 150 rpm shaking incubation for 48 h. After that, the mixture was centrifuged at 3000× *g* for 15 min. The resultant supernatant was concentrated into 80 mL using a rotary vacuum evaporator. The extract was lyophilized through freeze drying (−80 °C, 72 h), eventually yielding 12.25% (Figure 1A). The acquired powder was stored at −20 °C for future use with a voucher specimen number of CAE-202103. In addition, metformin was obtained from Sigma-Aldrich (St. Louis, MO, USA) and used as a positive control.

### 2.2. HPLC Fingerprinting Analysis

The CAE dissolved in 50% methanol was analyzed by an Agilent 1260 high-performance liquid chromatography (HPLC) system (Agilent Technologies, Wilmington, DE, USA), which consisted of a bin pump, degasser, autosampler, column oven, and diode array detector (DAD), at a wavelength of 300 nm. An Agilent Eclipse XDB-C18 column (4.6 × 250 mm, 5 μm) was used at RT. The samples were eluted using H_2_O and CH_3_CN with a gradient solvent increasing from 20% to 100% CH_3_CN for 45 min at a 1 mL/min flow rate. Paeonol (Glentham Life Sciences, Wiltshire, UK), one known single component derived from *Cynanchum atratum*, was used as an internal reference for CAE. Finally, the chromatograms were obtained from Agilent ChemStation software (Figure 1B,C).

### 2.3. Animal Experimental Design and Ethics Approvement

Thirty C57BL/6j mice (male, 6 weeks old) were purchased from Daehan Biolink (Gyeonggi-do, Korea). After 1 week of acclimatization at an RT of 20 ± 2 °C and 40–60% humidity with free access to a normal chow diet, the animals were randomly divided into five groups. The normal group was given a chow diet, and the other groups were administered a high-fat diet (HFD, D12494, 60% kcal% fat, Research Diet, New Brunswick, NJ, USA) in combination with fructose (20% *w/v*, dissolved in distilled water, Sigma-Aldrich, Darmstadt, Germany). Both food and water were provided ad libitum for ten weeks. Based on the actual clinical dosage (7.5–15 g/day/60 kg) and yield of extraction (12.25%), the dosage of CAE in the present study was calculated using a conversion formula according to U.S. Food and Drug Administration (FDA) guidance [22]. Metformin, an FDA-approved anti-diabetes agent, was applied as a positive control drug in the present study due to its widespread use for relieving various metabolic disorders in clinics. From the fifth week, CAE (100, 200 mg/kg/day) or metformin (100 mg/kg/day) were intragastrically provided separately to three groups for six weeks. In the meantime, only distilled water was administered via gavage once a day in the non-drug treatment groups (normal and HFHFD groups) (Figure 1D).

The total body weight, and food and water intakes were recorded weekly, and whole blood was collected from the abdominal aorta under ketamine anesthesia on the final day of the experiment. The complete blood count (CBC) was directly measured using an Exigo Eos Veterinary hematology analyzer (Boule Medical AB, Spanga, Sweden). The liver and abdominal fat tissue were removed and weighed. Then, liver tissues were fixed in 10% neutral formalin or stored in RNAlater solution (Ambion, Austin, TX, USA) or directly at −70 °C for further examination.

The present study was approved by the Institutional Animal Care and Use Committee of Daejeon University (approval number: DJUARB-2021006) and performed according to the Guide for Care and Use of Laboratory Animals (National Institutes of Health publication, 1996).

### 2.4. Fasting Blood Glucose Determination

Before sacrifice, mouse tail vein blood was collected via puncturing. Within 10 s, fasting blood glucose was determined with glucose test strips using an ACCU-CHEK Instant glucometer (Roche Diabetes Care GmbH, Mannheim, Germany).

### 2.5. Biochemistry Determination in Serum, Feces, and Liver Tissue

After 1 h of blood clotting, serum was separated from whole blood via 3000× *g* centrifugation for 15 min at 4 °C. Serum aspartate transaminase (AST), alanine transaminase (ALT), triglycerides (TG), total cholesterol (TC), low-density lipoprotein (LDL), and high-density lipoprotein (HDL) were measured using a Chemistry Auto Analyzer (Chiron, Emeryville, CA, USA). Meanwhile, hepatic and fecal TG and TC levels were determined using commercial enzymatic assay kits (Asan Pharmaceutical Co., Seoul, Korea). Free fatty acid (FFA) in the serum was analyzed quantitatively using an automatic biochemistry analysis system (AU480, Beckman coulter, Brea, CA, USA).

### 2.6. Histopathological Examination and Immunofluorescence in Liver Tissue

Formalin-fixed liver tissue was embedded with paraffin wax using a Leica embedding station (EG 1160, Nussloch, Germany) and sectioned into 5 μm thick sections with a microtome (Leica RM2235, Nussloch, Germany). Hematoxylin and eosin-stained liver sections were mounted using Canada balsam. Fresh liver tissues were embedded using FSC22 clear frozen section media (Leica, Germany) in liquid nitrogen. Silicone-coated slide sections (5 μm) were stained with oil red O solution and hematoxylin. For hepatic immunofluorescence determination, the frozen liver sections were washed with Tris-buffered saline (TBS) and blocked with 5% normal chicken serum for 60 min. Then, the anti-NF-kB p65 antibody (Supplementary Materials, Table S2) was used for incubation overnight. Finally, the secondary antibody conjugated with Alexa Fluor 488 (Supplementary Materials, Table S2) was further employed for 2 h of incubation. The fluorescent dye-stained liver sections were mounted using an aqueous mounting medium (Biomeda corp., Foster City, CA, USA). The Olympus inverted fluorescent microscope (IX71, Tokyo, Japan) and digital camera (DP70, Tokyo, Japan) were utilized to examine and photograph the liver tissue. For the objective evaluation of the histopathological findings, the NAFLD activity score (NAS) was used to estimate the grade of severity [23], and the percentage of fatty regions stained with oil red O and NF-kB p65 nuclear positivity was calculated using ImageJ (1.52a, National Institutes of Health, Bethesda, MD, USA).

### 2.7. Inflammation-Related Cytokines Measurement

According to the manufacturer’s instructions, the hepatic levels of tumor necrosis factor-α (TNF-α) and interleukin-10 (IL-10) were determined using enzyme-linked immunosorbent assay (ELISA) kits (BioLegend, San Diego, CA, USA for TNF-α; BD Biosciences, San Jose, CA, USA for IL-10). The protein concentration was quantified with a Pierce™ BCA Protein Assay Kit (Thermo Fisher Scientific, Lafayette, CO, USA). All of the results of the ELISAs were normalized according to the protein content (pg/mg protein). The TNF-α to IL-10 ratio was also calculated for evaluation of the inflammation degree.

### 2.8. Real-Time PCR Analysis 

Total RNA was extracted from the hepatic tissue with a QIAzol Lysis reagent according to the kit instructions. Then, cDNA was synthesized with a High-Capacity cDNA Reverse Transcription Kit (Thermo Fisher Scientific, San Jose, CA, USA). PCR amplification reactions were conducted with each primer (Supplementary Materials, Table S1) with a Rotor-Gene Q (Qiagen, Hilden, Germany) and Sybr Green kit. The PCR reaction was performed in a total reaction volume of 20 μL, consisting of the PCR mix, 1 μL of cDNA, and gene-specific primers (10 pmol each). The relative gene expression was represented by 2^−ΔCt^, using b-actin as a housekeeping gene for normalization.

Fresh stool samples were collected on the final experimental day and stored at −70 °C for DNA extraction. Stool microbial genomic DNA was extracted using the QIAamp DNA stool mini kit (Qiagen, Hilden, Germany). The purity and concentration of the isolated DNA samples were determined with a NanoDrop™ (Thermo Scientific). Real-time PCR was performed on a Rotor-Gene Q (Qiagen, Hilden, Germany) using a PowerUp SYBR Green Master Mix (Applied Biosystems, Lafayette, CO, USA). The Gram-positive and Gram-negative bacterial primers sequences are also presented in the Supplementary Materials, Table S1. PCR amplification reactions were carried out by following our previously used method [24]. Calculations of 2^−ΔCt^ reflect the relative bacterial abundance. The final results are expressed as the normalized fold change relative to the normal group.

### 2.9. Western Blot Analysis

Mice liver tissues were homogenized in RIPA buffer (Abcam, Cambridge, UK). The supernatant was isolated, and total protein concentrations were measured using a BCA kit (Thermo Scientific Scientific, San Jose, CA, USA). Prepared proteins were separated in 10% polyacrylamide gel via electrophoresis and then transferred to polyvinylidene fluoride (PVDF) membranes using the Mini-PROTEAN Tetra Cell System (BioRad, Hercules, CA, USA). The membranes were blocked using 3% bovine serum albumin (BSA), washed three times with Tween 20, and incubated with a primary antibody overnight at 4 °C (Supplementary Materials, Table S2). Subsequently, membranes were incubated with horseradish peroxidase-conjugated IgG for two hours. An advanced enhanced chemiluminescence (ECL) reagent (Thermo Fisher Scientific, San Jose, CA, USA) was used to screen the target protein. The final band on the membrane was detected using a FUSION Solo Image system (Vilber Lourmat, Marne-la-Vallée, France). The gray values were compared using ImageJ (1.52a, National Institutes of Health, Bethesda, MD, USA).

### 2.10. Endotoxin Determination

Fecal endotoxin was determined using the Pierce Chromogenic Endotoxin Quant Kit (A39552, Thermo Scientific, Rockford, IL, USA). Briefly, frozen fecal samples were diluted 1000-fold with endotoxin-free water. After 3000× *g* and 5 min of centrifugation, the 50 μL supernatants or standards (0–0.5 EU/mL) were mixed with the amebocyte lysate reagent and incubated in a 96-well plate at 37 °C for 8 min. Then, 100 μL of pre-warmed chromogenic substrate solution was added and subsequently placed in an incubator at 37 °C for 6 min. Finally, 50 μL of 25% acetic acid was added as a stop solution; the endpoint absorbance of the mixture was measured at 405 nm and calculated using the standard curve. 

### 2.11. Statistical Analysis

All data were expressed as the mean ± standard deviation (SD) and analyzed using the Statistical Package for the Social Sciences (SPSS, version 19.0, Chicago, IL, USA) software. Statistical significance of difference was determined using a one-way ANOVA, followed by the least significant difference (LSD) post hoc test. A *p*-value of less than 0.05 refers to a statistically significant result.

## 3. Results

### 3.1. CAE Reduced Surplus Body and Organ Weights, but Had No Relationship with Food and Fructose Intake

**Figure 2:**
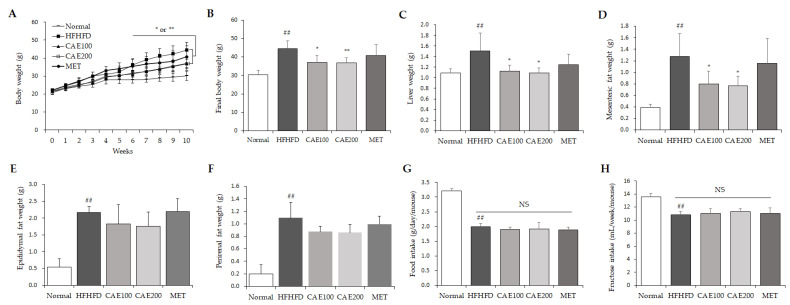
CAE counteracted excessive body, liver, and mesenteric fat mass but did not influence a high-fat diet and high-fructose intake itself. The body weight (**A**,**B**) and food (**G**)/fructose (**H**) intake were recorded once a week throughout the experiment. Liver mass (**C**), mesenteric (**D**), epididymal (**E**), and perirenal (**F**) adipose tissue weights were recorded on the final day (n = 6). ^##^ *p* < 0.01, as compared to normal group; * *p* < 0.05 and ** *p* < 0.01, as compared to the HFHFD group.

From the sixth week, a significant difference in body weight was observed between the HFHFD and the CAE group (*p* < 0.05 or *p* < 0.01, Figure 2A). At the end of the experiment, the body, liver, and mesenteric, epididymal, perirenal fat weights of the HFHFD mice were significantly higher than those of the normal mice (*p* < 0.01, Figure 2B–F). CAE treatment markedly reduced the elevation of these weights compared to the HFHFD control in a dose-dependent manner (*p* < 0.01, Figure 2B–D), but no significant reduction in epididymal, perirenal fat weights was found (Figure 2E,F). However, CAE did not influence the average high-fat diet and high-fructose intake compared to the other HFHFD-fed groups (Figure 2G,H). 

### 3.2. CAE Ameliorated Serum Biochemical Parameters and Fasting Blood Glucose Level

HFHFD treatment significantly increased the serum levels of AST, ALT, TG, TC, LDL, and FFA and markedly reduced serum HDL (*p* < 0.05 or *p* < 0.01, Table 1). However, CAE treatment notably attenuated excessive serum levels of AST, ALT, TG, TC, LDL, and FFA and noticeably restored the HDL level in serum compared to the HFHFD group (*p* < 0.05 or *p* < 0.01, Table 1). In addition, the level of fasting blood glucose (FBG) was markedly increased by the HFHFD compared to the level in the normal group. However, CAE treatment significantly reduced the fasting blood glucose level compared to the treatment of the HFHFD control.

### 3.3. CAE Reduced Lipid Accumulation and NAFLD Activity Score in Liver Tissue

**Figure 3:**
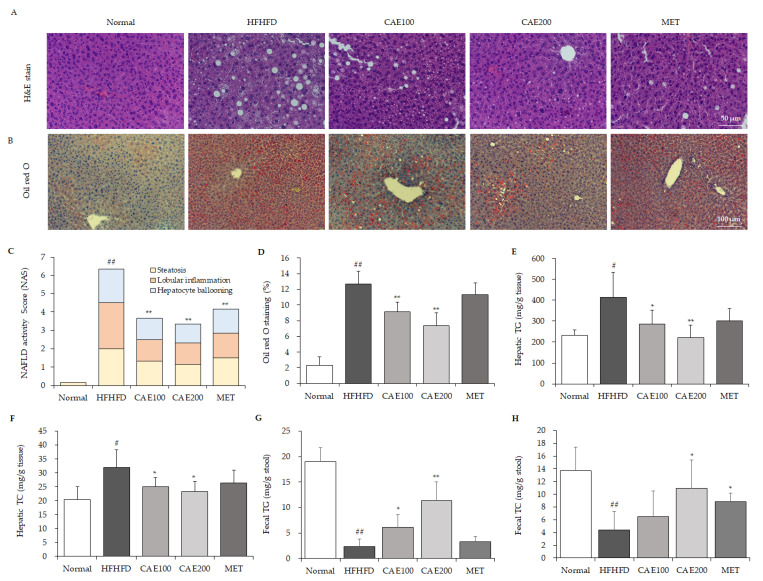
CAE inhibits lipid accumulation in liver tissue. The hepatic tissues were stained with H&E (**A**) and oil red O (**B**) separately (n = 6). The NAFLD activity score (**C**), including steatosis, lobular inflammation, and hepatocyte ballooning scores, was applied to compare the NAFLD grade. ImageJ software was used to quantify lipid accumulation from findings of oil red O staining (**D**). The hepatic and fecal triglycerides (**E**,**G**) and total cholesterol (**F**,**H**) values were determined using commercial enzymatic assay kits (Asan Pharmaceutical Co., Seoul, Korea) (n = 6). ^#^ *p* < 0.05 and ^##^ *p* < 0.01, as compared to normal group; * *p* < 0.05 and ** *p* < 0.01, as compared to the HFHFD group.

H&E-stained liver histological observation showed that an HFHFD induced plenty of fat vacuoles and obvious hepatocyte swelling compared to a normal diet, whereas CAE markedly improved these histopathological alterations (Figure 3A). The highest NAFLD activity score (NAS) was calculated in the HFHFD group. However, CAE and metformin treatment significantly reduced the NAS compared to the HFHFD control (*p* < 0.01, Figure 3C). Oil red O staining observation also indicated that lipid drops were extensively deposited in the liver tissue in the HFHFD group (Figure 3B). Nevertheless, CAE significantly reduced the lipid accumulation compared to the HFHFD control in a dose-dependent manner, and metformin did not affect lipid accumulation (Figure 3D). In accordance with the above histopathological findings, both hepatic TG and TC were distinctly elevated by HFHFD treatment. These excessive hepatic TG and TC levels were noticeably reduced by CAE rather than metformin (*p* < 0.05 or *p* < 0.01, Figure 3E,F). Interestingly, an HFHFD treatment markedly reduced the fecal TG and TC level compared to the normal diet treatment. In contrast, CAE treatment significantly enhanced the contents of fecal TG and TC compared to the HFHFD control (*p* < 0.05 or *p* < 0.01, Figure 3G,H).

### 3.4. CAE Stabilized the HFHFD-Induced Platelet and Leucocyte Alteration

The CBC results showed that an HFHFD significantly increased the platelet count and markedly lowered the WBC count (*p* < 0.05, Table 2) without showing changes in RBC and hemoglobin levels. In detail, an HFHFD markedly decreased the count of lymphocytes (*p* < 0.05, Table 2), but not monocytes and granulocytes. CAE noticeably reduced the platelet count and the platelets to lymphocytes ratio (PLR) and, meanwhile, distinctly inhibited the lessening of leucocyte numbers, especially lymphocytes, but not monocytes and granulocytes (*p* < 0.05 or *p* < 0.01, Table 2). Moreover, metformin also produced a pattern that was similar to CAE.

### 3.5. CAE Attenuated the Hepatic Inflammation Associated with Reduction in Endotoxin

**Figure 4:**
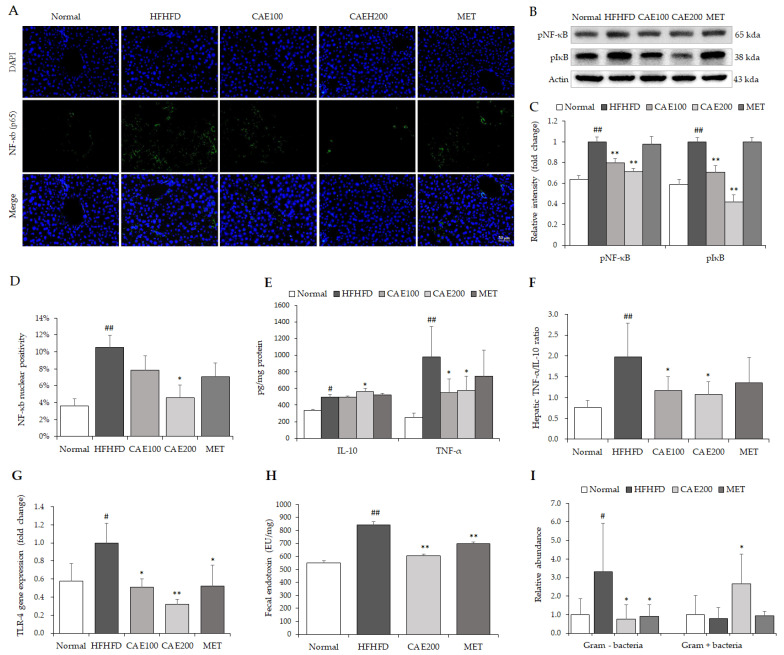
CAE attenuated hepatic inflammation-related factors. NF-Kb phosphorylation in liver tissues was detected using immunofluorescent staining (**A**) (n = 6). The quantification of NF-κb was performed using ImageJ software (**D**). The levels of hepatic phospho-NF-κB and p65 phospho-IκB were assessed via Western blot (**B**) (n = 6). The relative intensity was calculated using ImageJ software for comparison of fold change (**C**). Commercial ELISA kits determined serum anti-inflammatory (IL-10) and pro-inflammatory (TNF-α) cytokines (**E**), and the hepatic TNF-α to IL-10 ratio was calculated to evaluate the liver inflammation status (**F**) (n = 6). The gene expression of TLR-4 in liver tissue was assessed using real-time PCR (**G**) (n = 6). The endotoxin in feces was measured using a Chromogenic Limulus Amebocyte Lysate (LAL) Endotoxin Assay Kit (**H**) (n = 6). Both the relative abundance of Gram-negative and Gram-positive (**I**) bacteria in feces were also detected using corresponding specific primers (n = 6). ^#^ *p* < 0.05 and ^##^ *p* < 0.01, as compared to normal group; * *p* < 0.05 and ** *p* < 0.01, as compared to the HFHFD group.

As a crucial inflammation-related transcription factor, both hepatic NF-κB (p65) and IκB were perceptibly phosphorylated by the HFHFD treatment (Figure 4A–D). In contrast, CAE visibly attenuated the phosphorylation of NF-κB (p65) and IκB in the liver tissue compared to the HFHFD control (Figure 4A–D). Moreover, CAE significantly reduced the TNF-α and increased the IL-10 levels in the liver tissue compared to the normal levels. Additionally, CAE notably lowered the ratio of TNF-α to IL-10 in the liver tissue compared to the HFHFD control (*p* < 0.05, Figure 4E,F). Interestingly, an HFHFD markedly increased the endotoxin level and abundance of Gram-negative bacteria in the feces compared to the normal levels (*p* < 0.05 or *p* < 0.01, Figure 4H,I). However, CAE noticeably reduced both fecal endotoxin and Gram-negative bacteria abundance. On the contrary, CAE significantly enhanced Gram-positive bacteria abundance compared to the HFHFD control (*p* < 0.05, Figure 4I).

### 3.6. CAE Suppressed Lipogenesis and Accelerated Fatty Acid Oxidation-Related Genes and Proteins Expression in Liver Tissue

**Figure 5:**
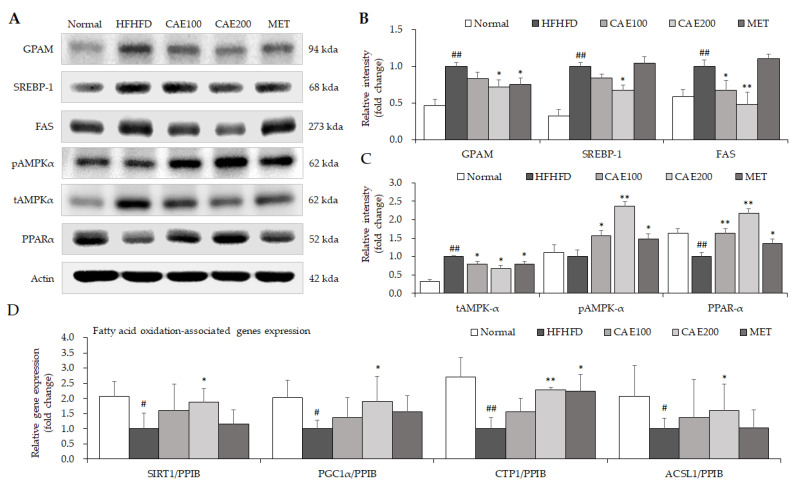
CAE ameliorated lipid metabolism through the alteration of lipid genesis and fatty acid oxidation. The levels of hepatic lipogenesis and fatty acid oxidation-related proteins were evaluated using Western blot (**A**) (n = 6). The relative intensity was calculated using ImageJ software for the comparison of fold change (**B**,**C**). The gene expression of fatty acid oxidation-related genes was determined via real-time PCR (**D**) (n = 6). ^#^ *p* < 0.05 and ^##^ *p* < 0.01, as compared to normal group; * *p* < 0.05 and ** *p* < 0.01, as compared to HFHFD group.

As expected, HFHFD treatment resulted in a significant elevation in hepatic glycerol-3-phosphate acyltransferase (GPAM), sterol regulatory element-binding protein 1 (SREBP-1), and fatty acid synthase (FAS) protein levels, and a noticeable decrease in the hepatic peroxisome proliferator-activated receptor (PPARα) protein level. Treatment using CAE notably inhibited the excessive protein levels of GPAM, SREBP-1, and FAS in the liver tissue (*p* < 0.05 or *p* < 0.01, Figure 5A,B). Meanwhile, CAE treatment more significantly incited the phosphorylation of hepatic AMPKα than the positive control (metformin, Figure 5A,C). In addition, CAE also obviously increased the hepatic protein level of PPARα. A series of β-oxidation-related gene expressions, including Sirtuin 1 (SIRT1), PPARγ-peroxisome proliferator-activated receptor gamma co-activator 1 alpha (PGC1α), carnitine acyltransferase 1 (CTP1), and adipose acyl-CoA synthetase-1 (ACSL1), was markedly up-regulated by CAE administration compared to the HFHFD control (*p* < 0.05 or *p* < 0.01, Figure 5D).

## 4. Discussion

Due to the fact that it has the most phenotypic similarity to clinic cases, the HFHFD-induced rodent model of NAFLD was extensively applied to investigate drug efficacy in preclinical studies [25]. The long-term consumption of high-fat and high-fructose diets can more strongly induce metabolic dysfunctions, such as hyperglycemia, hyperlipidemia, and insulin resistance, than a high-fat or high-fructose diet alone [26]. Meanwhile, the combined consumption of a high-fat, high-fructose diet could markedly induce obesity and liver steatosis with low-grade inflammation and hepatocyte injury [27]. In our study, the consumption of an HFHFD for ten weeks significantly increased the body weight of mice, mainly from the liver weight and mesenteric fat weight, even in low-intake conditions (Figure 2A–F). CAE treatment inhibited these aberrant elevations without a noticeable reduction in food consumption. In particular, only fat mass around the intestine was significantly reduced by CAE administration (Figure 2C) rather than other fat portions (perinephric and epididymal fat, Figure 2E,F). Unlike other portions of fats, mesenteric fat directly releases free fatty acid through the portal vein to the liver [28]. Therefore, the significant decrease in the mesenteric fat contributed to lower TG synthesis and lipid accumulation in the liver. Interestingly, CAE markedly changed the relative abundance of Gram-positive and Gram-negative bacteria in feces. This means that the overall structure of gut bacteria was regulated by the CAE treatment. Nevertheless, gut microbiota can be involved in the regulation of adipogenesis [29]. A conceivable explanation is that the fat reduction by CAE is associated with the intestinal commensal bacteria change. 

Usually, liver damage is mild in HFHFD models. However, in the present study, an obvious hepatic injury was still found in the HFHFD group, as evidenced by histopathologic alterations in the liver tissue, such as the existence of Mallory–Denk bodies and hepatocyte ballooning (Figure 3A–C), and the notable elevation of serum AST and ALT levels (Table 1), which are specific biochemical markers for liver injury. However, CAE ameliorated lobular inflammation and hepatocyte swelling in the liver tissue in line with serum transaminase levels (Figure 3A,C and Table 1). As we know, TNF-α and IL-10 are the major pro- and anti-inflammatory cytokines, respectively, playing vital roles in NAFLD progression [30,31]. The TNF-α/IL-10 ratio is also used as a marker of inflammatory response in clinic settings [32]. In keeping with histopathological improvement, CAE significantly reduced the hepatic TNF-α level and TNF-α/IL-10 ratio and markedly increased the level of IL-10 (Figure 4E,F). NF-kB is a major transcription factor that brings about the gene expression of the various pro-inflammatory cytokines [33]. As a prototypic NF-κB-IκB complex, the degradation of IκB leads to NF-κB activation following the signal transduction [34]. A high-fat diet can accelerate NF-kB signaling, whereas blocking the NF-kB ameliorates the high-fat-diet-induced fatty liver condition [35]. Moreover, PPARα exerts anti-inflammatory properties in the liver via the transrepression pathway [36]. CAE also suppresses the inflammatory response in the liver by increasing the level of hepatic PPARα. Thus, CAE alleviates liver injury by reducing hepatic inflammation, a mechanism that is mostly mediated by the inhibition of the NF-kB signaling pathway and the activation of PPARα (Figure 4A,D and Figure 5A). In addition, PGC1α4, a specific isoform of PGC1α, attenuates hepatic apoptosis through the regulation of hepatic inflammatory signaling [37]. Although we found that CAE significantly up-regulated the gene expression of PGC1α in hepatic tissue (Figure 5D), the gene isoforms were not investigated in the present study.

Endotoxin, also known as lipopolysaccharide, is a potent inflammatory inducer derived from an exterior membrane of Gram-negative bacteria [38]. It was proven that a high-fat diet accelerates endotoxin generation by changing the structure of the gut microbiota [39]. Excessive endotoxin leads to systematic inflammation through TLR-4, a natural receptor for LPS [39,40]. The anti-inflammatory capacity of CAE is probably caused by a noteworthy reduction in endotoxin and its receptor TLR-4 (Figure 4G,H), which are involved in the diminution of Gram-negative bacteria and the growth of Gram-positive bacteria (Figure 4I). 

Previous clinical studies revealed that thrombocytosis is found in obese subjects with chronic inflammation and patients with metabolic syndrome [41,42]. Moreover, moderate and severe NAFLD patients have higher platelet numbers in their peripheral blood than mildly affected patients [43]. Therefore, HFHFD-induced metabolic dysfunction with chronic inflammation might be a conceivable reason for thrombocytosis. Interestingly, long-term high-fat diet treatment caused leukopenia and TNF-α increase in mice [44]. In the present study, HFHFD significantly induced leukopenia, mainly due to the decrease in lymphocytes (Table 1). Furthermore, the platelet–lymphocyte ratio (PLR) is frequently used to indicate the degree of inflammation in clinic [45]. Nevertheless, CAE markedly lowered the excessive platelet counts, including PLR, and normalized the leukocyte numbers in accordance with the anti-inflammation properties.

In addition, without hyperphagia, an HFHFD can noticeably increase lipid flow and diminish the oxidation of fatty acid, which contributes to the formation of fatty liver [46]. Previous reports also showed that a high-fructose diet interferes with lipid metabolism by accelerating fat accumulation in the liver [47]. In our histopathological observation, HFHFD-induced hepatic lipid accumulation was demonstrated by oil red O overstaining and numerous lipid vacuoles in liver tissue (Figure 3A,B). In addition, the disruption of TG and TC homeostasis has been shown to induce lipid accumulation in the liver [48,49]. The results suggest that CAE markedly relieves diet-induced hepatosteatosis, as evidenced by reducing hepatic TG and TC levels, the degree of lipid accumulation in the liver, and the NAS scores (Figure 3A–F and Table 2). On the other hand, the fecal TG and TC levels revealed that CAE promotes lipid release via feces excretion as well (Figure 3G,H). Moreover, CAE significantly elevated the uncoupling protein 1 (UCP1) level in brown adipose tissue, contributing to energy expenditure through thermogenesis (Supplementary Materials, Figure S1). Hence, enhancing lipid excretion and energy expenditure might be the potential causes of attenuating lipid accumulation in the liver and mesenteric fat. 

Although relieving chronic inflammation in the liver is one of the immediate mechanisms that contributes to the prevention of NAFLD progression [50], other lipid metabolism-related mechanisms also need to be investigated. Accordingly, serval key proteins and genes were evaluated to explore potential molecular mechanisms. AMPK plays a vital role in regulating lipid homeostasis [51]. After phosphorylation, AMPK was shown to improve glucose uptake and lipid oxidation in various tissues, including the liver [51]. As an AMPK activator, the idea that metformin could be used as a first-line therapy for controlling blood glucose has been supported [52]. A high level of glucose can induce lipid accumulation through epigenetic regulation [53]. Metformin was shown to significantly attenuate lipid and glucose serum levels in humans and mice with NAFLD [54,55]. Therefore, this study used metformin as a positive agent. Remarkably, CAE boosted the AMPK activation more drastically than even metformin (Figure 5A). This finding is in keeping with the improvement of fasting blood glucose and lipid accumulation that CAE was shown to facilitate. 

In addition, SREBP-1 is a pivotal transcription factor for enhancing hepatic lipogenesis-related genes expressions, such as FAS and GPAM [56]. Both FAS and GPAM are vital catalyzing enzymes that contribute to lipogenesis via synthesizing fatty acids and glycerolipid, respectively [57,58]. These gene expressions reflected the fact that CAE ameliorated hepatic steatosis by inhibiting lipogenesis, probably mediated by hepatic SREBP-1, FAS, and GPAM (Figure 5A–C). Moreover, PPARs, fatty acid-regulated transcription factors, play a central role in regulating lipid metabolism and inflammation responses [59]. The specific knockout of hepatic PPARα impeded fatty acid catabolism, leading to lipid accumulation in the liver tissue [60]. Thus, CAE elevates the β-oxidation of fatty acids via the up-regulation of hepatic PPARα, as evidenced by a significant decline in the free fatty acid serum level. Diet-derived fatty acids contribute to the formation of TG, which is stored in lipid droplets, and the oxidation of fatty acid in mitochondria provides energy to hepatocytes [61]. Thus, the blockage of fatty acid oxidation leads to lipid accumulation in the liver. Further, as a co-activator, PGC1α can enhance the transcriptional activity of PPARα [62]. The overexpression of SIRT1 brought about the deacetylation of the PPARα and PGC1α complex [63], thereby increasing mitochondrial fatty acid oxidation and energy expenditure [64,65]. Besides, CTP1 is an enzyme that can catalyze mitochondria beta-oxidation in the liver [66]. These key lipid catabolism gene expressions, including SIRT1, PGC1α, CTP1, and ACSL1, showed how CAE accelerated hepatic fatty acid oxidation (Figure 5D). Consequently, we suggest that CAE exerts an anti-fatty liver effect both by reducing lipid generation and increasing fatty acid oxidation. 

## 5. Conclusions

In conclusion, the present study suggests that *Cynanchum atratum* can ameliorate a fatty liver by balancing lipid metabolism and enhancing fatty acid oxidation. Additionally, *Cynanchum atratum* reduces hyperglycemia and hyperlipidemia and alleviates the hepatic inflammation related to a reduction in endotoxin generated by Gram-negative bacteria in the gut. The activation of AMPK and PPARα likely mediates these effects. Therefore, these findings provide a reliable clue that can be used in new anti-NAFLD drug development.

## Figures and Tables

**Table 1:** Blood and serum biochemistry parameters.

Contents		Normal	HFHFD	CAE		MET
				100	200	
Serum	AST (IU/L)	51.0 ± 5.1	81.4 ± 13.4 ^##^	65.3 ± 7.4 *	62.4 ± 9.7 *	71.0 ± 14.6
	ALT (IU/L)	17.3 ± 3.0	48.2 ± 17.2 ^#^	31.6 ± 16.2	20.3 ± 4.9 *	44.1 ± 31.7
	TG (mg/dL)	116.9 ± 11.4	137.4 ± 16.9 ^#^	140.7 ± 16.8	122.7 ± 8.4 *	112.5 ± 10.8 **
	TC (mg/dL)	101.2 ± 5.6	180.9 ± 9.9 ^##^	169.4 ± 18.6	148.6 ± 22.4 *	162.5 ± 11.6 *
	LDL (mg/dL)	15.8 ± 0.6	27.8 ± 2.6 ^##^	22.7 ± 1.9 **	23.5 ± 1.2 **	26.3 ± 1.8
	HDL (mg/dL)	114.0 ± 14.3	72.5 ± 3.9 ^##^	114.4 ± 9.3 **	124.2 ± 13.6 **	113.8 ± 6.2 **
	FFA (mEq/L)	0.89 ± 0.12	1.03 ± 0.01 ^#^	0.92 ± 0.04 **	0.85 ± 0.16 *	0.97 ± 0.23
FBG (mg/dL)		88.5 ± 7.4	163.2 ± 40.6 ^##^	106.3 ± 10.9 *	119.0 ± 18.8 *	130.7 ± 16.8 *

^#^*p* < 0.05 and ^##^ *p* < 0.01, as compared to normal group; * *p* < 0.05 and ** *p* < 0.01, as compared to the HFHFD group; HFHFD, high-fat and high-fructose diet; CAE, *Cynanchum atratum* extract; MET, metformin; AST, aspartate aminotransferase; ALT, alanine aminotransferase; TG, triglyceride; TC, total cholesterol; HDL, high-density lipoprotein; LDL, low-density lipoprotein; FFA, free fatty acid; FBG, fasting blood glucose.

**Table 2:** Analysis of cell blood counts.

Hematology Indexes (n = 6)	Normal	HFHFD	CAE		MET
			100	200	
Erythrocytes (10^9^/mL)	7.88 ± 0.69	8.06 ± 0.28	8.04 ± 0.36	8.00 ± 0.41	8.68 ± 0.74
Hemoglobin (g/dL)	12.33 ± 1.11	12.37 ± 0.53	12.49 ± 0.53	12.31 ± 0.71	13.73 ± 1.96
Platelets (10^6^/mL)	321 ± 203	584 ± 240 ^#^	456 ± 244	281 ± 173 *	329 ± 219
PLR (ratio)	31.04 ± 17.75	206.53 ± 149.61 ^#^	96.38 ± 89.48	46.79 ± 50.60 *	45.26 ± 42.47 *
Leukocytes (10^6^/mL)	11.56 ± 4.51	6.56 ± 1.59 ^#^	9.37 ± 2.70	10.89 ± 3.01 *	11.41 ± 4.04 *
Lymphocytes (10^6^/mL)	8.04 ± 2.83	4.33 ± 1.82 ^#^	6.64 ± 2.44	8.40 ± 2.27 **	8.80 ± 3.19 *
Monocytes (10^6^/mL)	0.74 ± 0.43	0.45 ± 0.05	0.60 ± 0.16	0.61 ± 0.21	0.70 ± 0.24 *
Granulocytes (10^6^/mL)	2.79 ± 1.36	1.78 ± 0.23	2.12 ± 0.56	1.87 ± 0.73	1.91 ± 0.71

^#^*p* < 0.05, as compared to normal group; * *p* < 0.05 and ** *p* < 0.01, as compared to the HFHFD group; HFHFD, high-fat and high-fructose diet; CAE, *Cynanchum atratum* extract; MET, metformin; PLR, platelets to lymphocytes ratio.

## Data Availability

The data that support the findings of this study are available within the article.

## Supplementary Materials

The following are available online at https://www.mdpi.com/article/10.3390/cells11010023/s1, Table S1: Primer sequences used for real-time PCR, Table S2: Antibodies information for Western blot and immunofluorescence, Figure S1: Expression of UCP1 protein in brown adipose tissue.
