# Supplementary material for: Cynanchum atratum Alleviates Non-Alcoholic Fatty Liver by Balancing Lipogenesis and Fatty Acid Oxidation in a High-Fat, High-Fructose Diet Mice Model
Source: Cells. 2021 Dec 22;11(1):23. doi: 10.3390/cells11010023 (PMC8750091; doi:10.3390/cells11010023)
Supplement: Supplementary file 1 [file cells-11-00023-s001.zip › cells-1482546-supplementary.pdf]

Supplementary tables and figure

Table S1. Primer sequences used for real-time PCR.

Name	Primer sequence	OAT	Reference
Gram-negative bacterium	Forward: 5′- AYG ACG TCA AGT CMT CAT GG -3′<br>Reverse: 5′- AAC TGG AGG AAG GTG GGG AY -3′	65.0	[1]
Gram-positive bacerium	Forward: 5′- AGGAGGTGATCCAACCGC -3′<br>Reverse: 5′- GAYGACGTCAARTCMTCATGC -3′	60.0	[1]
TLR4	Forward: 5′- GGAAGGACTATGTGATGTGACC -3′<br>Reverse: 5′- GCTCTTCTAGACCCATGAAATTGG -3′	59.9	[2]
CPT1α	Forward: 5′- CATGATTGCAAAGATCAATCGG -3′<br>Reverse: 5′- CTTGACATGCGGCCAGTG -3′	57.1	[3]
ACSL1	Forward: 5′- CCAGAAGGGCTTCAAGACTG -3′<br>Reverse: 5′- GCCTTCTCTGGCTTGTCAAC -3′	62.0	[4]
PGC1α	Forward: 5′- AGCCGTGACCACTGACAACGAG -3′<br>Reverse: 5′- GCTGCATGGTTCTGAGTGCTAAG -3′	57.9	[5]
SIRT1	Forward: 5′- GCTGACGACTTCGACGACG -3′<br>Reverse: 5′- TCGGTCAACAGGAGGTTGTCT -3′	60.7	[6]
PPIB	Forward: 5′- TCCATCGTGTCATCAAGGACTT -3′<br>Reverse: 5′- CTCATCTGGGAAGCGCTCA -3′	59.0	[3]

Abbreviations: TLR4, toll-like receptor 4; CPT1α, carnitine palmitoyltransferase 1 alpha; ACSL1, adipose acyl-CoA synthetase-1; PGC1α, Peroxisome proliferator-activated receptor-gamma coactivator 1 alpha; SIRT1, Sirtuin 1; PPIB, Peptidylpropyl isomerase B; OAT, optimized annealing temperature.

Table S2. Antibodies information for western blot and immunofluorescence.

Peptide/Protein target	Manufacturer	Catalog	Dilution ratio
Actin	Thermo Fisher scientific	MA 5-11869	1:1,000
GPAM	abcam	ab69990	1:1,000
PPAR-α	abcam	ab24509	1:1,000
Total AMPK-α	Cell signaling technology	#5831	1:1,000
Phospho AMPK-α	Cell signaling technology	#2535	1:1,000
FAS	Cell signaling technology	#3180	1:1,000
SREBP-1	abcam	ab28481	1:1,000
UCP1 (4E5)	Santa Cruz Biotechnology	sc-293418	1:1,000
Phospho-NF-κB p65 (Ser536)	Cell signaling technology	#3033	1:1,000
Phospho IkB	Invitrogen	MA5-16161	1:1,000
Goat Anti-Rabbit IgG (HRP)	GeneTex	GTX213110-01	1:5,000
Goat Anti-Mouse IgG (HRP)	GeneTex	GTX213111-01	1:5,000
Anti-NF-kB p65 (phospho S536)	abcam	Ab86299	1:200
Goat Anti-Rabbit IgG H&L (Alexa Fluor® 488)	abcam	ab150077	1:400

Abbreviations: GAMP, Glycerol-3-Phosphate Acyltransferase, Mitochondrial; PPAR- α, Peroxisome proliferator-activated receptor; AMPK, AMP-activated protein kinase; FAS, fatty acid synthase; SREBP-1, Sterol regulatory element-binding protein 1; UCP1, uncoupling protein 1; HRP, horseradish peroxidase; IgG, Immunoglobulin G.

Figure S1. Expression of UCP1 protein in brown adipose tissue.

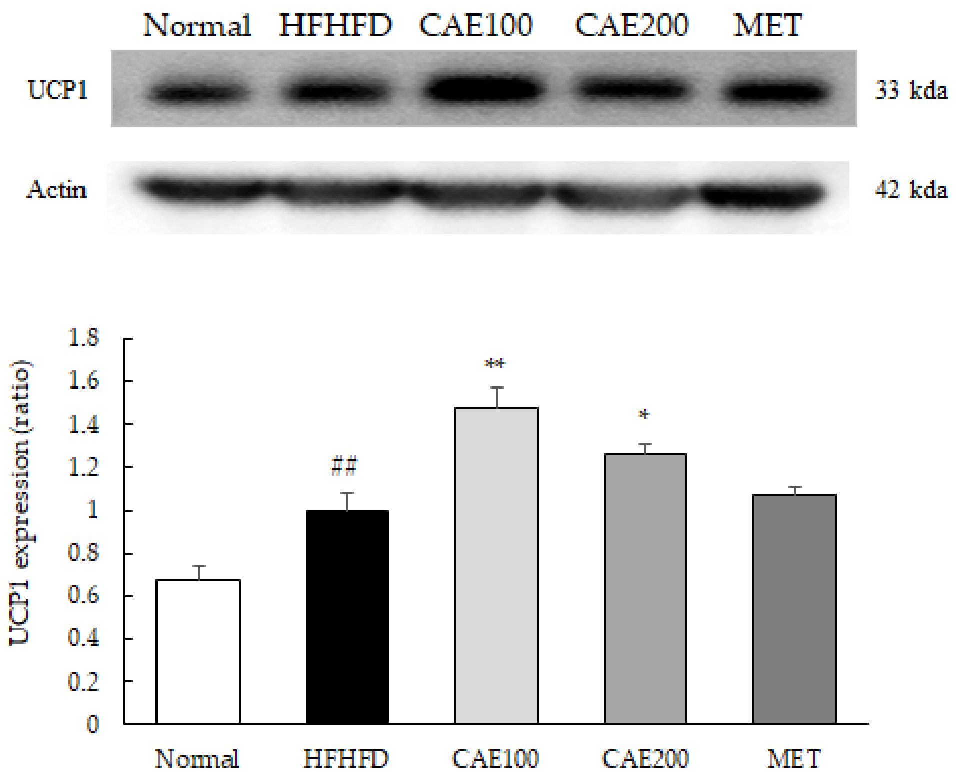

Supplementary Figure legend

Figure S1. Expression of UCP1 protein in brown adipose tissue.
The Uncoupling Protein-1 (UCP-1) protein levels in brown adipose tissue were evaluated using Western blot (n = 6). The relative intensity was calculated using ImageJ software for comparison of fold change. $^{##}p < 0.01$, as compared to normal group; $^{*}p < 0.05$, $^{**}p < 0.01$ as compared to HFHFD group.

Reference

1. Wang, J.-H.; Bose, S.; Shin, N.R.; Chin, Y.-W.; Choi, Y.H.; Kim, H. Pharmaceutical Impact of Houttuynia Cordata and Metformin Combination on High-Fat-Diet-Induced Metabolic Disorders: Link to Intestinal Microbiota and Metabolic Endotoxemia. **2018**, *9*, doi:10.3389/fendo.2018.00620.
2. Liu, S.; Gallo, D.J.; Green, A.M.; Williams, D.L.; Gong, X.; Shapiro, R.A.; Gambotto, A.A.; Humphris, E.L.; Vodovotz, Y.; Billiar, T.R. Role of toll-like receptors in changes in gene expression and NF-kappa B activation in mouse hepatocytes stimulated with lipopolysaccharide. *Infection and immunity* **2002**, *70*, 3433-3442, doi:10.1128/iai.70.7.3433-3442.2002.
3. Warfel, J.D.; Vandanmagsar, B.; Dubuisson, O.S.; Hodgeson, S.M.; Elks, C.M.; Ravussin, E.; Mynatt, R.L. Examination of carnitine palmitoyltransferase 1 abundance in white adipose tissue: implications in obesity research. *American journal of physiology. Regulatory, integrative and comparative physiology* **2017**, *312*, R816-r820, doi:10.1152/ajpregu.00520.2016.
4. Fortis-Barrera, M.d.l.Á.; Alarcón-Aguilar, F.J.; Becerril-García, A.; Flores-Sáenz, J.L.E.; Almanza-Pérez, J.C.; García-Lorenzana, M.; Lazzarini-Lechuga, R.C.; Román-Ramos, R.; Blancas-Flores, G. Mechanism of the Hypoglycemic Activity and Hepatoprotective Effect of the Aqueous Extract of Cecropia obtusifolia Bertol. *Journal of Medicinal Food* **2019**, *23*, 783-792, doi:10.1089/jmf.2019.0126.
5. Lee, W.J.; Kim, M.; Park, H.S.; Kim, H.S.; Jeon, M.J.; Oh, K.S.; Koh, E.H.; Won, J.C.; Kim, M.S.; Oh, G.T., et al. AMPK activation increases fatty acid oxidation in skeletal muscle by activating PPARalpha and PGC-1. *Biochemical and biophysical research communications* **2006**, *340*, 291-295, doi:10.1016/j.bbrc.2005.12.011.
6. Buler, M.; Aatsinki, S.M.; Skoumal, R.; Hakkola, J. Energy sensing factors PGC-1α and SIRT1 modulate PXR expression and function. *Biochemical pharmacology* **2011**, *82*, 2008-2015, doi:10.1016/j.bcp.2011.09.006.
